# Value of ^18^F–FDG PET/CT for predicting EGFR mutations and positive ALK expression in patients with non-small cell lung cancer: a retrospective analysis of 849 Chinese patients

**DOI:** 10.1007/s00259-017-3885-z

**Published:** 2017-11-21

**Authors:** Zhilei Lv, Jinshuo Fan, Juanjuan Xu, Feng Wu, Qi Huang, Mengfei Guo, Tingting Liao, Shuqing Liu, Xiaoli Lan, Shanshan Liao, Wei Geng, Yang Jin

**Affiliations:** 1 Key Laboratory of Respiratory Diseases of the Ministry of health, Department of Respiratory and Critical Care Medicine, Union Hospital, Tongji Medical College, Huazhong University of Science and Technology, 1277 Jiefang Avenue, Wuhan, 430022 China; 2 Department of Nuclear Medicine, Union Hospital, Tongji Medical College, Huazhong University of Science and Technology, Wuhan, 430022 China; 3 Hubei Key Laboratory of Molecular Imaging, Union Hospital, Tongji Medical College, Huazhong University of Science and Technology, Wuhan, 430022 China; 4 Biobank, Union Hospital, Tongji Medical College, Huazhong University of Science and Technology, Wuhan, 430022 China

**Keywords:** Epidermal growth factor receptor, Anaplastic lymphoma kinase, Mutation, Non-small cell lung cancer, Positron emission tomography, Standard uptake value

## Abstract

**Purpose:**

Epidermal growth factor receptor (EGFR) mutations and the anaplastic lymphoma kinase (ALK) rearrangement are the two most common druggable targets in non-small cell lung cancer (NSCLC). However, genetic testing is sometimes unavailable. Previous studies regarding the predictive role of ^18^F–FDG PET/CT for EGFR mutations in NSCLC patients are conflicting. We investigated whether or not ^18^F–FDG PET could be a valuable noninvasive method to predict EGFR mutations and ALK positivity in NSCLC using the largest patient cohort to date.

**Methods:**

We retrospectively reviewed and included 849 NSCLC patients who were tested for EGFR mutations or ALK status and subjected to ^18^F–FDG PET/CT prior to treatment. The differences in several clinical characteristics and three parameters based on ^18^F–FDG PET/CT, including the maximal standard uptake value (SUV_max_) of the primary tumor (pSUV_max_), lymph node (nSUV_max_) and distant metastasis (mSUV_max_), between the different subgroups were analyzed. Multivariate logistic regression analysis was performed to identify predictors of EGFR mutations and ALK positivity.

**Results:**

EGFR mutations were identified in 371 patients (45.9%). EGFR mutations were found more frequently in females, non-smokers, adenocarcinomas and stage I disease. Low pSUV_max_, nSUV_max_ and mSUV_max_ were significantly associated with EGFR mutations. Multivariate analysis demonstrated that pSUV_max_ < 7.0, female sex, non-smoker status and adenocarcinoma were predictors of EGFR mutations. The receiver operating characteristic (ROC) curve yielded area under the curve (AUC) values of 0.557 and 0.697 for low pSUV_max_ alone and the combination of the four factors, respectively. ALK-positive patients tended to have a high nSUV_max_. Younger age and distant metastasis were the only two independent predictors of ALK positivity.

**Conclusion:**

We demonstrated that low pSUVmax is associated with mutant EGFR status and could be integrated with other clinical factors to enhance the discriminability on the EGFR mutation status in some NSCLC patients whose EGFR testing is unavailable.

## Introduction

Over the last decade, the introduction of tyrosine-kinase inhibitors (TKIs) has enabled a remarkable paradigm shift in the treatment of non-small cell lung cancer (NSCLC), especially in advanced adenocarcinoma (ADC). Epidermal growth factor receptor (EGFR) mutations and the echinoderm microtubule-associated protein-like 4 (EML4)-anaplastic lymphoma kinase (ALK) rearrangement are the two most-prevalent druggable targeting categories in NSCLC patients [1]. Randomized clinical trials have demonstrated that progression-free survival (PFS) is longer with TKIs than with chemotherapy when EGFR mutations [2, 3] or the ALK rearrangement [4, 5]are present in advanced NSCLC. Furthermore, the TKI efficacy is dependent on the presence of EGFR mutations or the ALK rearrangement. These discoveries have led to the recommendation of molecular profiling as the standard of care for advanced ADC patients [6, 7]. However, the acquisition of sufficient good-quality tumor tissues for gene alteration analyses remains challenging in many cases of advanced NSCLC.

^18^F–FDG PET is a widely used noninvasive diagnostic modality that is based on different rates of ^18^F–FDG uptake. EGFR signaling regulates the glucose metabolic pathway in EGFR-mutated lung cancer cells, and EGFR-TKIs decrease lactate production and glucose consumption [8]. Another study has also shown that EGFR-TKIs reverse the Warburg effect and decrease ^18^F–FDG uptake in mice bearing H1975- EGFR mutant or H1993-EGFR mutant tumors [9]. Thus, ^18^F–FDG avidity on PET may be useful as a noninvasive biomarker for predicting EGFR mutations and the ALK rearrangement.

Previous data concerning the association between ^18^F–FDG uptake and EGFR mutations are conflicting [10–18], and little is known about the correlation between ^18^F–FDG avidity and the ALK rearrangement [19, 20]. Thus, this study retrospectively reviewed patients in the last 5 years and included 849 NSCLC patients to investigate whether or not ^18^F–FDG PET could be a valuable method for predicting EGFR mutations and the ALK rearrangement in NSCLC.

## Materials and methods

### Patients and inclusion criteria

We retrospectively reviewed all NSCLC patients whose EGFR or ALK statuses were analyzed and who underwent PET/CT from January 2012 to September 2016. A total of 1042 patients was identified. We excluded 193 patients from this study for one of three reasons: (1) for 43 patients, the time interval between sampling and PET/CT exceeded 1 month; (2) 26 patients had a history of malignancy; and (3) 124 patients had been treated before sampling for the gene alteration analysis. Hence, a total of 849 patients were ultimately included in this study. Patient clinical characteristics including age, sex, smoking history, histopathology, tumor size, nodal involvement, distant metastasis and tumor stage were recorded. Non-smokers were defined as patients who never smoked or smoked less than 100 cigarettes in their lifetimes. The rest were categorized as ever-smokers. Tumor node metastasis (TNM) staging was based on the IASLC 8th TNM Lung Cancer Staging System.

### ^18^F–FDG PET/CT acquisition and analysis

PET/CT was performed on a Discovery LS PET/CT system (GE Medical Systems). Patients fasted for at least 6 h before examination. The blood glucose concentration was tested and confirmed to be less than 6.6 mmol/L before intravenous injection of 5.5 MBq/kg ^18^F–FDG. Imaging acquisition was conducted 1 h after ^18^F–FDG administration. Attenuation correction CT was performed using the following parameters: 120 kV, 80 mA, and 4.25 mm collimation. Then the PET scan was immediately acquired from the head to the upper leg in 2-dimensional mode at 3 min per bed position. Typically, 6–8 bed positions were examined depending on the height of patient. The PET data were reconstructed using the ordered set expectation maximization algorithm method. CT images were used for attenuation correction of the PET data and anatomic localization. The co-registered images were then displayed on the Xeleris Workstation (GE Medical System) for evaluation.

All PET/CT data were independently reviewed by two experienced nuclear medicine physicians. All reviewers were blinded to the EGFR and ALK status. A region of interest (ROI) was placed over the primary tumor, nodal metastasis and distant metastasis to measure each SUV_max_. SUV_max_ was calculated with the most commonly applied formula: SUV_max_ = maximum pixel activity/ (injected dose/body weight).

### ALK Ventana immunohistochemistry (IHC)

Ventana IHC is a fully automated IHC assay that is based on a monoclonal antibody, D5F3. Ventana IHC has been approved by the US FDA and China FDA for the identification of patients with NSCLC who are eligible for treatment with ALK TKIs. According to the manufacturer’s instructions and scoring algorithm, the assay was conducted with 4 μm-thick formalin-fixed, paraffin-embedded tissue sections. The result was dichotomous whereby the presence of any percentage of positive tumor cells with strong granular cytoplasmic staining was deemed ALK positive, while all other observations were deemed ALK negative.

### EGFR mutation analysis

EGFR mutations were analyzed based on the principle of the amplification refractory mutation system (ARMS). Briefly, resected, aspirated or biopsied primary tumor, lymph node, or distant metastasis samples or pleural effusion samples were fixed in 10% neutral buffered formalin and embedded in paraffin wax. The DNA was extracted from the formalin-fixed, paraffin-embedded tissue sections using the QIAamp DNA FFPE tissue kit (Qiagen NV, Venlo,Netherlands) according to the manufacturer’s instructions. Polymerase chain reaction was carried out with the Mx3000PtM (Stratagene, La Jolla, USA) using the EGFR 29 Mutations Detection Kit (Amoy Diagnostics, Xiamen, People’s Republic of China), and the result was interpreted according to the manufacturer’s instructions.

### IHC analysis

The IHC analysis was conducted by pathologists from the Department of Pathology of Wuhan Union Hospital. Briefly, the 4 μm-thick formalin-fixed, paraffin-embedded tissue sections were analyzed using the following primary antibodies: TTF-1 (clone 8G7G3/1, Maixin, Fuzhou, China), NapsinA (multiclone, Maixin), CK-7 (clone OV-TL 12/30, Maixin), and Ki67 (clone MX006, Maixin). Positive expression of TTF-1 was defined as >5% of tumor cells with distinct cytoplasmic or nuclear staining. The presence of >50% of tumor cells with diffuse and intense cytoplasmic staining was deemed positive for Napsin-A and CK-7. The percentage of tumor cells with distinct nuclear staining of Ki67 was denoted with a Ki67 score.

### Statistical analysis

Clinical characteristics including the PET/CT parameters were compared according to the EGFR or ALK status using the chi-squared test and Student’s *t*-test. A two-sided *p* value < 0.05 was defined as statistically significant. Receiver operating characteristics (ROC) curves were constructed to obtain the cutoff value of the primary tumor SUV_max_ (pSUV_max_) for predicting the EGFR mutations status. Logistic regression analysis was performed to identify independent predictors of the EGFR or ALK status. Clinical parameters and a pSUV_max_ with *p* < 0.05 in the univariate analysis, as well as previously reported factors associated with EGFR or ALK status, were further analyzed by multivariate regression analysis. Variates with *p* < 0.05 in the multivariate analysis were deemed independent predictors, and the odds ratios and 95% confidence intervals of the predictors were obtained. ROC curves were constructed for the combined independent factors for predicting mutant EGFR. All analyses were performed using the SPSS software package (version 16.0; SPSS, Chicago, IL, USA).

## Results

### Patient and tumor characteristics

Among the 849 NSCLC patients tested for EGFR and ALK status in our hospital between January 2012 to September 2016, 808 were tested for EGFR, 223 were tested for ALK, and 182 were tested for both. The clinical characteristics are summarized in Table 1 based on whether the patients were tested for EGFR or ALK.

**Table 1:** Association between clinical characteristics and the EGFR and ALK status in NSCLC

Characteristics	EGFR Mutant	EGFR Wild-Type	Total	p value	ALK Positive	ALK Negative	Total	*p* value
Age (years), Mean ± SD (range)	58.7 ± 9.8 (30–83)	58.7 ± 10.5 (25–85)	58.7 (25–85)	0.908	50.5 ± 9.7 (31–69)	58.8 ± 9.6 (29–85)	58.2 ± 9.8 (29–85)	0.001
Sex				<0.001				0.615
Male	165 (35.3%)	303 (64.7%)	468 (57.9%)		8 (6.6%)	114 (93.4%)	122 (54.7%)	
Female	206 (60.6%)	134 (39.4)	340 (42.1%)		9 (8.9%)	92 (91.1%)	101 (45.3%)	
Smoking status				<0.001				0.299
Never smoker	278 (55.8%)	220 (44.2%)	498 (61.6%)		13 (9.4%)	126 (90.6%)	139 (62.3%)	
Ever smoker	93 (30%)	217 (70%)	310 (38.4%)		4 (4.8%)	80 (93.5%)	84 (37.7%)	
Tumor size, Mean ± SD	3.2 ± 1.6	3.7 ± 2.0		0.021	4.2 ± 2.6	3.8 ± 2.1		0.445
pSUV_max_, Mean ± SD	8.7 ± 4.8	10.7 ± 6.2		<0.001	9.4 ± 4.2	10.4 ± 5.4		0.745
nSUV_max_, Mean ± SD	7.3 ± 4.2	9.3 ± 5.5		<0.001	10.6 ± 3.5	8.6 ± 4.9		0.091
mSUV_max_, Mean ± SD	8.0 ± 4.9	9.5 ± 5.7		0.005	9.9 ± 6.1	8.8 ± 6.1		0.575
Nodal involvement				0.030				0.652
0	149 (52.1%)	137 (47.9%)	286 (35.4%)		5 (6.7%)	70 (93.3%)	75 (33.6%)	
1	20 (43.5%)	26 (56.5%)	46 (0.57%)		0 (0.0%)	9 (100.0%)	9 (4.0%)	
2	74 (47.1)	83 (52.9%)	157 (19.4%)		3 (6.2%)	45 (93.8%)	48 (21.5%)	
3	128 (40.1%)	191 (59.9%)	319 (39.5%)		9 (9.9%)	82 (90.1%)	891 (40.8%)	
Metastasis				0.618				0.009
0	163 (47.0%)	184 (53.0%)	347 (42.9%)		2 (2.1%)	94 (97.9%)	96 (43.0%)	
1	208 (45.1%)	253 (54.9%)	461 (57.1%)		15 (13.4%)	112 (86.6%)	127 (57.0%)	
Stage				<0.001				0.060
I	87 (61.3%)	55 (38.7%)	142 (17.5%)		1 (2.5%)	39 (97.5%)	40 (17.9%)	
II	16 (32.7%)	33 (67.3%)	49 (6.4%)		0 (0.0%)	11 (100.0%)	11 (4.9%)	
III	61 (38.9%)	96 (61.1%)	157 (19.4%)		1 (2.2%)	44 (97.8%)	45 (20.2%)	
IV	207 (45.0%)	253 (55.0%)	460 (56.8%)		15 (11.8%)	112 (88.2%)	127 (57.0%)	
Histology				<0.001				0.083
Adenocarcinoma	367 (50.2%)	364 (49.8%)	731 (90.5%)		17 (8.9%)	173 (91.9%)	190 (85.2%)	
Non-adenocarcinoma	7 (9.1%)	70 (90.9%)	77 (9.5%)		0 (0.0%)	33 (100.0%)		
Squamous cell carcinoma	5 (8.6%)	53 (91.4%)	58 (7.2%)		0 (0.0%)	25 (100.0%)	25 (11.2%)	
Large cell carcinoma	1 (25%)	3 (75%)	4 (0.5%)		0 (0.0%)	1 (100.0%)	1 (0.4%)	
Undefined NSCLC	1 (6.7%)	14 (93.3%)	15 (1.9%)		0 (0.0%)	7 (100.0%)	7 (3.1%)	
TTF-1				<0.001				0.046
Negative	13 (14.1%)	79 (85.9%)	92 (17.2%)		0 (0.0%)	1541 (100.0%)	41 (19.8%)	
Positive	212 (47.7%)	232 (52.3%	444 (82.8%)		16 (9.9%)	150 (90.9%)	166 (80.2%)	
NaspinA				<0.001				0.037
Negative	15 (16.5%)	76 (83.5%)	91 (25.9%)		0 (0.0%)	44 (100.0%)	44 (27.3%)	
Positive	119 (45.8%)	141 (54.2%)	260 (74.1%)		12 (10.4%)	105 (89.6%)	117 (72.7%)	
CK7				0.005				0.362
Negative	2 (8.7%)	21 (91.3%)	23 (5.8%)		0 (0.0%)	15 (100.0%)	15 (10.1%)	
Positive	142 (37.9%)	233 (62.1%)	375 (94.2%)		14 (10.4%)	120 (89.6%)	134 (89.9%)	
Ki67 score, Mean ± SD	22.4 ± 21.2	33.4 ± 22.1		0.002	16.9 ± 9.6	38.4 ± 26.1		<0.001

Note: unless otherwise indicated, data in parentheses are percentages

Abbreviations: *NSCLC*, non-small-cell lung cancer; *EGFR*, epidermal growth factor receptor; *ALK*, anaplastic lymphoma kinase; *SD*, standard deviation; *SUV*_*max*_, maximal standard uptake value; *pSUV*_*max*_, primary tumor SUV_max_; *nSUV*_*max*_, nodal metastasis SUV_max_; *mSUV*_*max*_, distant metastasis SUV_max_

Of the 808 patients tested for EGFR status, EGFR mutations were identified in 371 (45.9%); the patient group included 340 women (42.1%) and 468 men (57.9%) with a median age of 58.7 years (range, 25–85), and 498 (63.6%) were non-smokers. Seven hundred and thirty-one patients (90.5%), 58 patients (7.2%) and 19 patients (2.3%) were histologically confirmed to have ADCs, squamous cell carcinomas and other subtypes, respectively; the other subtypes included four large cell carcinomas and 15 undefined NSCLCs. One hundred and forty-two (17.5%), 49 (6.4%), 157 (19.4%) and 460 (56.8%) patients had stage I, stage II, stage III and stage IV disease, respectively. The median SUV_max_ of the primary tumor was 9.8 (range, 0.8–45.7).

Of the 223 patients tested for ALK, 17 (7.6%) were positive for ALK; the patient group included 101 women (45.3%) and 122 men (54.7%) with a median age of 58.2 years (range, 29–85), and 139(62.3%) were non-smokers. One hundred and ninety (85.2%), 25 (11.2%) and eight (3.5%) patients had histologically confirmed ADCs, squamous cell carcinomas and other subtypes, respectively; the other subtypes included one large cell carcinoma and seven undefined NSCLCs. Forty (17.9%), 11 (4.9%), 45 (20.2%) and 127 (57.0%) patients had stage I, stage II, stage III and stage IV disease, respectively. The median SUV_max_ of the primary tumor was 10.4 (range, 0.8–33.2).

### Association between clinical characteristics and EGFR mutations

**Fig. 1:**
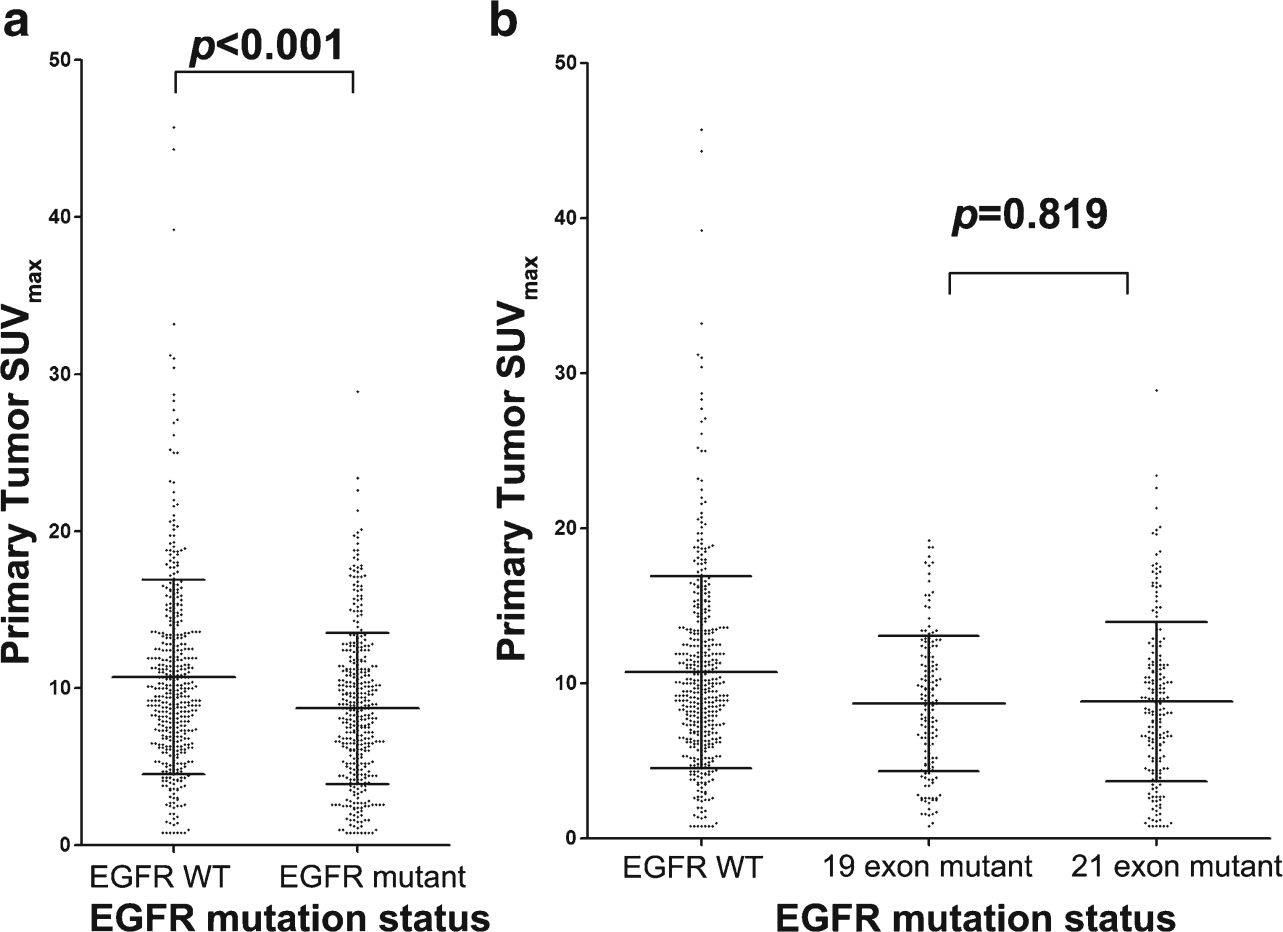
Comparison of non-small cell lung cancer ^18^F–FDG uptake according to EGFR mutation status. Primary tumor SUV_max_ are shown for subjects categorized according to epidermal growth factor receptor (EGFR) mutation status including wild-type EGFR (EGFR WT) and mutations in EGFR exon 19 and exon 21

**Fig. 2:**
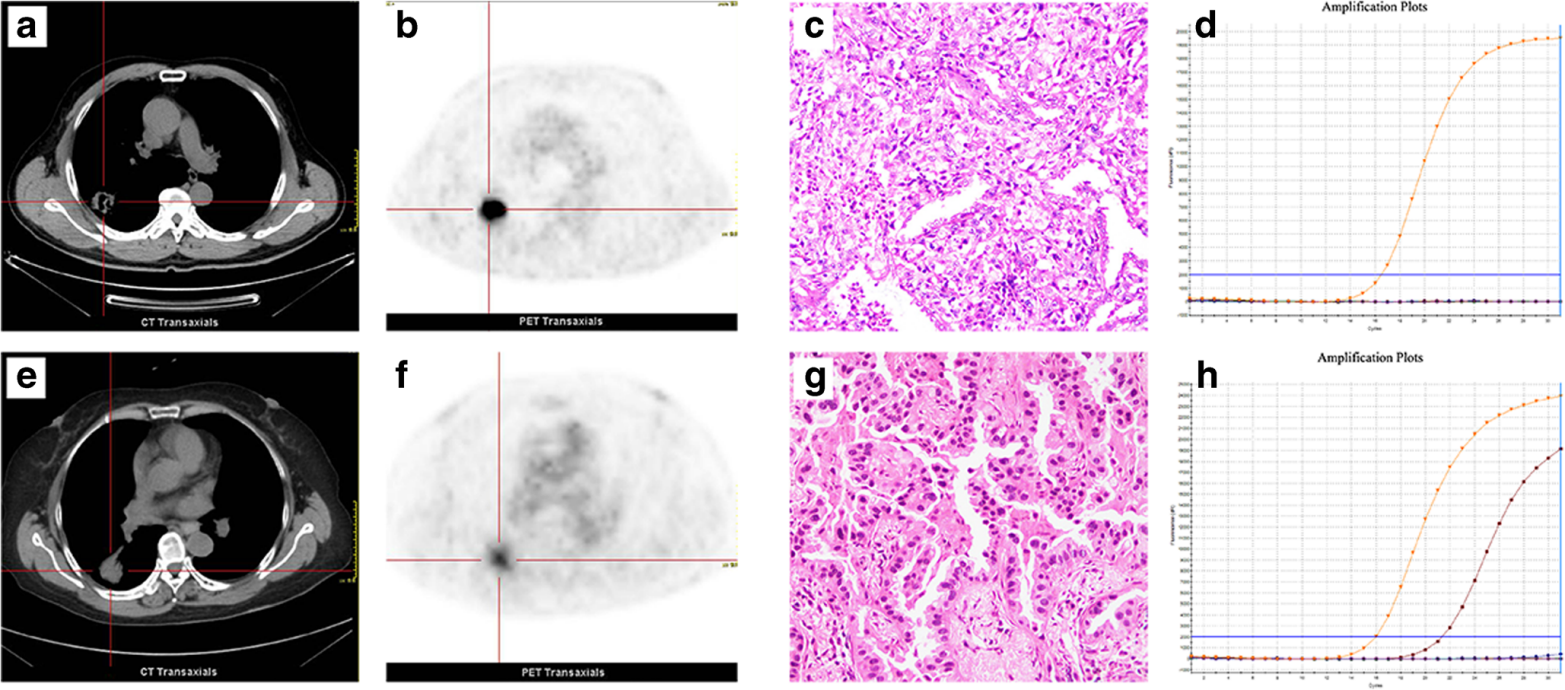
Representative EGFR status and ^18^F–FDG PET/CT findings. *Top panel*,findings of a 53-year-old man with EGFR-wild type lung adenocarcinoma. CT (**a**) and PET (**b**) images show a 2.9-cm-sized hypermetabolic mass in the right upper lobe (pSUV_max_ = 18.8). Hematoxylin-eosin staining (**c**) shows histological type of adenocarcinoma and the ARMS method (**d**) demonstrates wild type EGFR status. *Bottom panel*, findings of a 62-year-old woman with EGFR-mutant lung adenocarcinoma. CT (**e**) and PET (**f**) show a 2.6-cm-sized mass with mild ^18^F–FDG uptake (pSUV_max_ = 4.8) in the right lower lobe. Hematoxylin-eosin staining (**g**) shows histological type of adenocarcinoma and the ARMS method (**h**) demonstrates L858R substitution mutation in EGFR exon 21

The clinical characteristics of the NSCLC patients are summarized in Table 1 based on the EGFR status. EGFR mutations were found more frequently in women (60.6% vs. 35.3%; *p* < 0.001), non-smokers (55.8% vs. 30.0%; *p* < 0.001), ADCs (50.2% vs. 9.1%; *p* < 0.001), and stage I patients (61.3% vs. 32.7%, 38.9%, and 45.0%; *p* < 0.001). Positive expression of IHC marker TTF-1 (47.7% vs. 14.1%; *p* < 0.001), NaspinA (45.8% vs. 16.5%; *p* < 0.001), and CK7 (37.9% vs. 8.7%; *p* = 0.005) were significantly associated with EGFR mutations. The Ki67 scores (22.4 ± 21.2 vs. 33.4 ± 22.1; *p* = 0.002) were lower in the EGFR-mutant NSCLC patients than in the EGFR wild-type patients. The PET parameters of the pSUV_max_ (8.7 ± 4.8 vs. 10.7 ± 6.2; *p* < 0.001) (Fig. 1 a), nodal metastases SUV_max_ (nSUV_max_) (7.3 ± 4.2 vs. 9.3 ± 5.5; *p* < 0.001) and distant metastases SUV_max_ (mSUV_max_)(8.0 ± 4.9 vs. 9.5 ± 5.7; *p* = 0.005) were lower in the EGFR-mutant NSCLCs than in the EGFR wild-type NSCLCs. There were no differences in the pSUV_max_ results between the different EGFR mutation types, including in-frame deletion in exon 19 and substitution mutation in exon 21 (Fig. 1 b). Representative PET/CT images of two patients with EGFR mutant or wild-type NSCLC were shown (Fig. 2). The ROC curve analysis revealed that the cutoff point for the pSUV_max_ was 7.0; 72.8% sensitivity, 38.5% specificity, a 54.6% positive predictive value, a 58.2% negative predictive value, and 57.1% accuracy were achieved, and the area under curve (AUC) was 0.557 (95%CI, 0.517–0.596) with *p* = 0.001. Thus, although EGFR mutations were more frequently found in patients with pSUV_max_ < 7.0 (*p* = 0.001), pSUV_max_ was only a marginal significant predictor of EGFR mutations.

Due to the large differences in the EGFR mutation rates and ^18^F–FDG avidity between ADC and non-ADC, the ADC-only group was separately analyzed; the data are summarized in Table 2. The results were similar to those of the NSCLC group, except for CK7, which was not associated with EGFR mutations.

**Table 2:** Association between clinical characteristics and the EGFR and ALK status in adenocarcinoma

Characteristics	EGFR Mutant	EGFR Wild-Type	Total	*p* value	ALK Positive	ALK Negative	Total	*p* value
Age (years), Mean ± SD (range)	58.6 ± 9.8 (30–83)	58.0 ± 10.5 (25–85)	58.3 ± 10.1 (25–85)	0.367	50.5 ± 9.7 (31–69)	58.3 ± 9.5 (29–85)	57.6 ± 9.7 (29–85)	0.001
Sex				<0.001				0.803
Male	159 (39.4%)	245 (60.6%)	404 (55.3%)		8 (8.2%)	89 (91.8%)	97 (51.1%)	
Female	205 (62.7%)	122 (37.3)	327 (44.7%)		9 (9.7%)	84 (90.3%)	93 (48.9%)	
Smoking status				<0.001				0.589
Never smoker	275 (58.4%)	198 (41.6%)	473 (64.7%)		13 (10.2%)	115 (89.8%)	128 (67.4%)	
Ever smoker	89 (34.5%)	169 (65.5%)	258 (35.3%)		4 (6.5%)	58 (93.5%)	62 (32.6%)	
Tumor size, Mean ± SD	2.9 ± 1.8	3.1 ± 2.0		0.302	3.5 ± 2.1	3.6 ± 2.0		0.349
pSUV_max_, Mean ± SD	8.6 ± 4.7	10.0 ± 6.0		<0.001	9.4 ± 5.3	9.7 ± 4.8		0.852
nSUV_max_, Mean ± SD	7.3 ± 4.1	9.3 ± 5.5		<0.001	10.6 ± 3.5	8.2 ± 4.7		0.046
mSUV_max_, Mean ± SD	7.9 ± 5.0	9.4 ± 5.7		0.010	9.9 ± 6.1	8.2 ± 5.3		0.353
Nodal involvement				0.033				0.708
0	146 (55.7%)	116 (44.3%)	262 (35.8%)		5 (7.9%)	58 (92.1%)	63 (33.2%)	
1	20 (48.8%)	21 (51.2%)	41 (5.6%)		0 (0.0%)	7 (100.0%)	7 (3.7%)	
2	73 (52.1%)	67 (47.9%)	140 (19.2%)		3 (7.5%)	37 (92.5%)	40 (21.1%)	
3	125 (43.4%)	163 (56.6%)	288 (39.4%)		9 (11.3%)	71 (88.8%)	80 (42.1%)	
Metastasis				0.223				0.010
0	160 (52.5%)	145 (47.5%)	305 (41.7%)		2 (2.6%)	76 (97.4%)	78 (41.1%)	
1	204 (47.9%)	222 (52.1%)	426 (58.3%)		15 (13.4%)	97 (86.6%)	112 (58.9%)	
Stage				0.010				0.083
I	85 (62.5%)	51 (37.5%)	136 (18.6%)		1 (2.9%)	34 (97.1%)	35 (18.4%)	
II	16 (42.1%)	22 (57.9%)	38 (5.2%)		0 (0.0%)	7 (100.0%)	7 (3.7%)	
III	60 (45.5%)	72 (54.5%)	132 (18.1%)		1 (2.8%)	35 (97.2%)	36 (18.9%)	
IV	203 (47.8%)	222 (52.2%)	425 (58.1%)		15 (13.4%)	97 (86.6%)	112 (58.9%)	
TTF-1				<0.001				0.376
Negative	6 (13.6%)	38 (86.4%)	44 (9.1%)		0 (0.0%)	15 (100.0%)	15 (8.5%)	
Positive	212 (48.3%)	227 (51.7%)	439 (90.9%)		16 (9.9%)	145 (90.9%)	161 (91.5%)	
NaspinA				<0.001				0.363
Negative	10 (17.5%)	47 (82.5%)	57 (18.1%)		0 (0.0%)	15 (100.0%)	15 (22%)	
Positive	119 (46.1%)	139 (53.9%)	258 (81.9%)		12 (10.4%)	103 (89.6%)	115 (78%)	
CK7				0.225				1.000
Negative	0 (0.0%)	2 (100.0%)	2 (0.6%)		0 (0.0%)	1 (100.0%)	1 (0.8%)	
Positive	138 (39.4%)	212 (60.6%)	350 (99.4%)		14 (11.5%)	108 (88.5%)	122 (99.2%)	
Ki67 score, Mean ± SD	20.9 ± 18.8	31.1 ± 22.7		0.005	16.9 ± 9.6	31.9 ± 24.6		0.006

Note: unless otherwise indicated, data in parentheses are percentages

Abbreviations: *EGFR*, epidermal growth factor receptor; *ALK*, anaplastic lymphoma kinase; *SD*, standard deviation; *SUV*_*max*_, maximal standard uptake value; *pSUV*_*max*_, primary tumor SUV_max_; *nSUV*_*max*_, nodal metastasis SUV_max_; *mSUV*_*max*_, distant metastasis SUV_max_

### Association between clinical characteristics and ALK status

The ALK-positive NSCLC patients were more frequently younger age (50.5 ± 9.7 vs. 58.8 ± 9.6; *p* = 0.001). Positive ALK expression was observed only in the ADC patients (17/190 vs. 0/33), although the *p* value was 0.083 because the population of non-ADC patients was relatively small (Table 1). Although there was no significant difference in sex and smoking history between the ALK-positive and ALK-negative groups, females (8.9% vs. 6.6%) and non-smokers (9.4% vs. 4.8%) tended to have higher positivity rates than men and ever-smokers. Interestingly, positive expression of ALK was exclusively observed in TTF-1-, NaspinA- and CK7-positive NSCLC patients regardless of ADC or non-ADC status. The Ki67 scores (16.9 ± 9.6 vs. 38.4 ± 26.1; *p* = 0.026) were lower in the ALK-positive group than in the ALK-negative group. The nSUV_max_ was the only PET parameter that was higher in the ALK-positive patients than in the ALK-negative patients (10.6 ± 3.5 vs. 8.6 ± 4.9), with a marginal *p* value (0.091). The pSUV_max_ and mSUV_max_ were not significantly different between the two groups.

When the ADC group was separately analyzed, a young age (50.6 ± 11.4 vs. 58.9 ± 9.5; *p* < 0.001), high nSUV_max_ (10.7 ± 4.6 vs. 8.3 ± 5.2; *p* = 0.004) and low Ki67 score were significantly associated with positive ALK expression (Table 2).The other results were similar to those of the NSCLC groups.

### Prediction of the EGFR mutation status

**Fig. 3:**
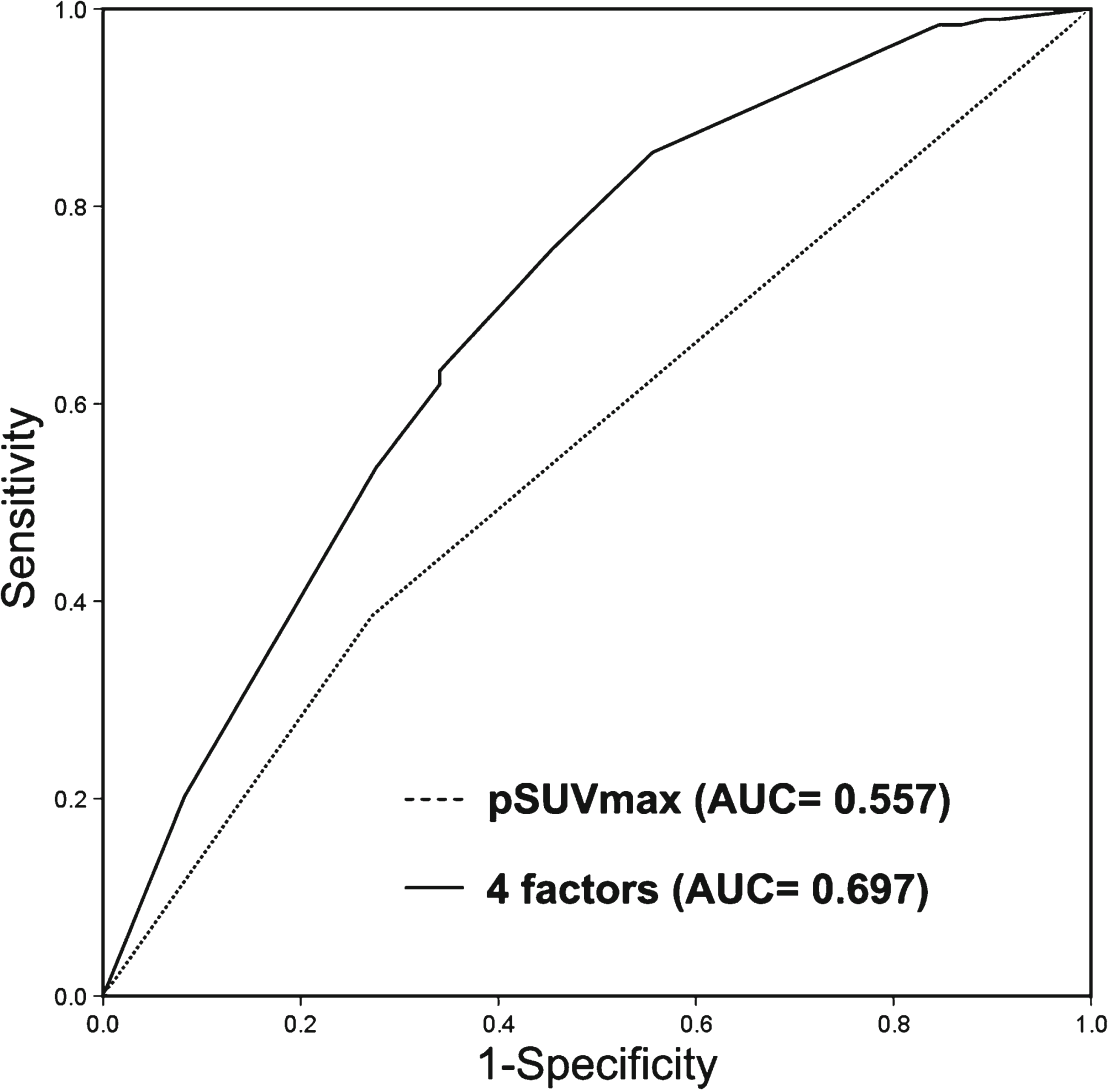
Receiver operating characteristic curves of primary tumor SUV_max_ and combination of four factors (pSUV_max_, sex, smoking history and histological type) for predicting EGFR mutation

For the NSCLC group (Table 3), the univariate logistic regression analysis showed that sex, smoking status, histology, pSUV_max_, tumor size, nodal involvement, distant metastasis, and tumor stage were significantly correlated with EGFR mutations. Then inclusion of these factors together in the multivariate regression analysis revealed that sex, smoking status, histology and pSUV_max_ remained independent variables for predicting EGFR mutations. Female sex (odds ratio [OR], 1.83; *p* = 0.003), non-smoker status (OR, 1.79; *p* = 0.006), ADC (OR, 7.09; *p* < 0.001) and pSUV_max_ < 7.0 (OR, 1.48; *p* = 0.041) were significant predictors of EGFR mutations. Additionally, a ROC curve analysis was conducted to evaluate the predictive value of these factors (Fig. 3), and the AUC of the categorical pSUV_max_ < 7.0 was 0.557. When the four criteria were used together, the AUC was 0.697.

**Table 3:** Univariate and multivariate analysis of various predictive factors for the EGFR status in NSCLC

Characteristics	Univariate Analysis OR (95% CI)	*p* value	Multivariate Analysis OR (95% CI)	*p* value
Age	1.00 (0.99–1.01)	0.908		
Sex		<0.001		0.003
Male	Reference		Reference	
Female	2.82 (2.12–3.77)		1.83 (1.23–2.73)	
Smoking status		<0.001		0.006
Never smoker	2.95 (2.18–3.98)		1.79 (1.18–2.72)	
Ever smoker	Reference		Reference	
Histology		<0.001		<0.001
Adenocarcinoma	9.92 (4.50–21.86)		7.09 (2.93–17.17)	
Non-adenocarcinoma	Reference		Reference	
Primary tumor SUV_max_	Reference	0.001		0.041
< 7.0	1.68 (1.25–2.26)		1.48 (1.02–2.16)	
≥ 7.0	Reference		Reference	
Tumor size	0.92 (0.86–0.99)	0.021		0.862
Nodal involvement		0.031		0.246
0				
1	0.71 (0.38–1.33)	0.279		
2	0.8 (0.56–1.21)	0.318		
3	0.62 (0.45–0.85)	0.003		
Distant metastasis		0.601		1.000
0	Reference			
1	0.93 (0.70–1.23)			
Stage		<0.001		0.347
I	Reference			
II	0.31 (0.15–0.60)	0.001		
III	0.40 (0.25–0.64)	<0.001		
IV	0.52 (0.35–0.76)	0.001		

Abbreviations: NSCLC, non-small-cell lung cancer; EGFR, epidermal growth factor receptor; SUV_max_, maximal standard uptake value; OR, odds ratio; CI, confidence interval

For the ADC-only group (Table 4), the univariate logistic regression analysis showed that sex, smoking status, pSUV_max_, tumor size, nodal involvement and tumor stage were associated with EGFR mutations. In the multivariate logistic regression analysis, female sex (OR, 1.91; *p* = 0.002), non-smoker status (OR, 1.74; *p* = 0.010), and pSUV_max_ < 7.0 (OR, 1.51; *p* = 0.036) were independent factors for predicting EGFR mutations in ADC patients.

**Table 4:** Univariate and multivariate analysis of various predictive factors for the EGFR status in adenocarcinoma

Characteristics	Univariate Analysis OR (95% CI)	*p* value	Multivariate Analysis OR (95% CI)	*p* value
Age	1.01 (0.99–1.02)	0.366		
Sex		<0.001		0.002
Male	Reference		Reference	
Female	2.59 (1.92–3.50)		1.91 (1.29–2.87)	
Smoking status		<0.001		0.010
Never smoker	2.64 (1.93–3.61)		1.74 (1.14–2.66)	
Ever smoker	Reference		Reference	
Primary tumor SUV_max_		0.006		0.036
< 7.0	1.53 (1.13–2.09)		1.51 (1.03–2.21)	
≥ 7.0	Reference		Reference	
Tumor size	0.96 (0.89–1.04)	0.302		
Nodal involvement		0.034		0.250
0				
1	0.76 (0.39–1.46)	0.407		
2	0.87 (0.57–1.31)	0.492		
3	0.61 (0.44–0.85)	0.004		
Metastasis		0.223		1.000
0	Reference			
1	0.83 (0.62–1.12)			
Stage		0.011		0.547
I	Reference			
II	0.44 (0.21–0.91)	0.026		
III	0.50 (0.31–0.81)	0.005		
IV	0.55 (0.37–0.82)	0.003		

Abbreviations: *EGFR*, epidermal growth factor receptor; *SUV*_*max*_, maximal standard uptake value; *OR*, odds ratio; *CI*, confidence interval

### Prediction for ALK status

For the NSCLC group (Table 5), which included 190 ADC patients and 33 non-ADC patients, univariate regression showed that a young age was the only statistically significant variate that was associated with positive ALK expression (OR, 0.92). Patients with distant metastasis also tended to be ALK positive (OR, 1.66; 95% CI, 0.98–2.81), although the *p* value was 0.058. In the multivariate analysis, sex, smoking history, histology, and the pSUV_max_ were analyzed together with age and distant metastasis. Younger age was still the only independent predictor of ALK positivity, although the OR of distant metastasis was 4.23 (*p* = 0.071). The pSUV_max_ was not associated with ALK status.

**Table 5:** Univariate and multivariate analysis of various predictive factors for the ALK status in NSCLC

Characteristics	Univariate Analysis OR (95% CI)	*p* value	Multivariate Analysis OR (95% CI)	*p* value
Age	0.92 (0.88–0.97)	0.001	0.93 (0.88–0.99)	0.014
Sex		0511		0.528
Male	Reference		Reference	
Female	1.39 (0.52–3.76)		1.51 (0.42–5.40)	
Smoking status		0.219		0.594
Never smoker	2.06 (0.65–6.6)		0.67 (0.16–2.88)	
Ever smoker	Reference		Reference	
Histology		0.117		0.998
Adenocarcinoma	5.13 (0.66–39.65)			
Non-adenocarcinoma	Reference			
Primary tumor SUV_max_	0.96 (0.87–1.06)	0.455	0.97 (0.86–1.10)	0.615
Nodal metastasis SUV_max_	1.08 (0.97–1.22)	0.173		
Distant metastasis SUV_max_	1.03 (0.94–1.13)	0.561		
Tumor size	1.09 (0.87–1.38)	0.445		
Nodal involvement		0.846		
0	Reference			
1	0.76 (0.17–3.37)	0.999		
2	0.933 (0.21–4.01)	0.927		
3	1.54 (0.49–4.80)	0.460		
Metastasis		0.058		0.071
0	Reference		Reference	
1	1.66 (0.98–2.81)		4.23 (0.89–20.20)	
Stage		0.234		
I	Reference			
II	1.53 (0.43–5.45)	0.508		
III	1.58 (0.62–4.08)	0.340		
IV	2.24 (0.98–5.11)	0.056		

Abbreviations: *NSCLC*, non-small-cell lung cancer; *ALK*, anaplastic lymphoma kinase; *SUV*_*max*_, maximal standard uptake value; *OR*, odds ratio; *CI*, confidence interval

The results of the separate analysis of the 190 ADC patients were similar to those of the NSCLC group (Table 6). Notably, the nSUV_max_ was not included the multivariate analysis because of the small sample size. Likewise, too few of the ADC patients were negative for TTF-1, NaspinA and CK7, so TTF-1, NaspinA and CK7 were also not included.

**Table 6:** Univariate and multivariate analysis of various predictive factors for the ALK status in adenocarcinoma

Characteristics	Univariate Analysis OR (95% CI)	*p* value	Multivariate Analysis OR (95% CI)	*p* value
Age	0.92 (0.90–0.95)	<0.001	0.93 (0.89–0.99)	0.014
Sex		0.174		0.583
Male	Reference		Reference	
Female	1.45 (0.85–2.48)		0.70 (0.19–2.54)	
Smoking status		0.213		0.647
Never smoker	1.45 (0.81–2.61)		1.41 (0.32–6.16)	
Ever smoker	Reference		Reference	
Primary tumor SUV_max_	1.02 (0.98–1.07)	0.332	0.97 (0.86–1.09)	0.615
Tumor size	1.04 (0.90–1.19)	0.611		
Nodal involvement		0.225		
0				
1	0.48 (0.06–3.76)	0.487		
2	1.58 (0.75–3.31)	0.225		
3	1.77 (0.94–3.33)	0.079		
Metastasis		0.158		0.071
0	Reference		Reference	
1	1.49 (0.86–2.58)		4.22 (0.88–20.0)	
Stage		0.235		
I	Reference			
II	1.99 (0.47–8.36)	0.350		
III	2.37 (0.87–6.42)	0.091		
IV	2.52 (1.04–6.11)	0.041		

Abbreviations: *ALK*, anaplastic lymphoma kinase; *SUV*_*max*_, maximal standard uptake value; *OR*, odds ratio; *CI*, confidence interval

## Discussion

TKIs have shown remarkable therapeutic effects and prolonged PFS in NSCLC patients with EGFR mutations or the ALK rearrangement [2–5]. These discoveries have led to the recommendation of molecular profiling as the standard of care for advanced NSCLC patients [6, 7]. However, the availability of sufficient good-quality tumor tissues for the gene alteration analyses is often challenging in advanced NSCLC patients. In this study, we demonstrate that NSCLC patients with EGFR mutations had lower pSUV_max_ measurements based on ^18^F–FDG PET than NSCLC patients with wild-type EGFR and that low pSUV_max_ could be integrated with other clinical factors to enhance the discriminability on the EGFR mutation status in some NSCLC patients whose EGFR testing is unavailable.

Data from previous studies that have investigated the value of ^18^F–FDG PET for predicting EGFR status are conflicting. Na et al. [18] and Cho et al. [21] revealed that a lower pSUV_max_ was an independent variate for predicting EGFR mutations. Two other groups reported the opposite result, where a higher pSUV_max_ predicted EGFR mutations [10, 14]. Moreover, multiple groups reported no association between the pSUV_max_ and EGFR status [12, 22].

Our data were consistent with observation by Na et al. [18]and Cho et al. [21], whereby a lower pSUV_max_ was an independent variate for predicting EGFR mutations. Cho et al. included 58 ADC and three non-ADC patients regardless of tumor stage; the study was conducted in Korea, and the EGFR mutation rate was 50%. The OR in the multivariate analysis that assessed the predictive value of a low pSUV_max_ for EGFR mutations was 12.97 (*p* = 0.005) [21]. The study by Na et al. contained 100 NSCLC patients, including 53 ADC and 47 non-ADC patients regardless of tumor stage; the study was also conducted in Korea, and the EGFR mutation rate was 21%. The OR of a low pSUV_max_ being predictive of EGFR mutations in the study by Na et al. was 1.3 (*p* = 0.025), which was similar to our result [18].

Several possible reasons may underlie these contradictory results. Our study included NSCLCs of all stages and histological types. In a separate analysis of stage IV ADC, the pSUV_max_ was not different between the EGFR-mutant and wild-type stage IV ADC patients, which was consistent with the result from Lee’s group [13]. Hence, the tumor stage and histological type of the studied patient population could significantly influence the results concerning this topic. Only two groups reported that a high pSUV_max_ was positively associated with EGFR mutations [10, 14]. However, all other studies revealed that EGFR-mutant patients tended to have a lower pSUV_max_ than EGFR wild-type patients, although the results were not statistically significant in the studies by Huang et al. [14] and Ko et al. [10]. Huang’s group included 77 stage III and stage IV ADC patients (excluding the bronchoalveolar subtype), and Ko’s group included 132 stage I-IV ADC patients who also had pretreatment serum CEA and CT assessments. The high EGFR mutation rate (64%) may suggest patient selection bias, and the patient population size in Huang’s study was relatively small. In Ko’s study, the requirement for pretreatment serum CEA and CT assessments may have promoted patient selection bias compared to other studies.

The results from the studies by Chung et al. [23] and Mak et al. [12] showed no statistically significant difference in the pSUV_max_ between the EGFR-mutant and wild-type patients. Lee et al. revealed that the pSUV_max_ was statistically significant only in the univariate regression analysis but not in the multivariate regression analysis. Our results showed that despite the statistically significant predictive role of pSUV_max_ for determining the EGFR mutation status, the pSUV_max_ measurements between the EGFR-mutant and wild-type groups substantially overlapped. Moreover, the AUC was only 0.557. The ADC patient population sizes were only 106, 90, and 135 in the studies by Chung et al., Mak et al. and Lee et al., respectively. Therefore, the small number of patients and modest discriminating power of the pSUV_max_ can explain these discrepant results.

Guan's group identified tumor size as a predictor for EGFR mutation on multivariate analysis, which is not consistent with our study. The higher proportion of non-ADC patients in Guan group’s data may cause this discrepancy. The primary tumor size of ADC patients tend to be smaller than that of non-ADC NSCLC patients. In our data, the average tumor size of ADC patients was 3.28 cm vs. 5.13 cm in non-ADC NSCLC patients. According to the cutoff value of 5 cm in Guan group’s study, the patients with primary tumor size >5 cm was 46.6% in non-ADC patients vs. 13.6% in ADC patients in our cohort. Thus, non-ADC NSCLC patient population composed much higher proportion patients with primary tumor size >5 cm and generally accepted much lower EGFR mutant rate than ADC patient population. Thus, the proportion of non-ADC patients in studied cohort will significantly affect the statistical result of the correlation between tumor size and EGFR mutation status. The proportion of non-ADC patients in Guan group’s study was 23.4% vs. 9.5% in our data. Actually, when ADC patients and non-ADC NSCLC patients were analyzed as a whole in our study, the primary tumor size of EGFR mutant patients was also smaller than that of EGFR wild-type patients (*p* = 0.02). However, there was no significant difference of tumor size between EGFR mutant patients and EGFR wild-type patients when ADC-only group was separately analyzed. Thus, the higher proportion of non-ADC NSCLC patients in Guan group’s data compared with our included population may cause this discrepancy.

In our study, the pSUV_max_ was associated with mutant EGFR status. The AUC was 0.697 when the four criteria, including pSUV_max_ < 7.0, female, non-smoker and histologic type of ADC were analyzed together for predicting EGFR mutation status. The result of the separate analysis of the ADC group was similar. An important notable issue is that the clinicians preferred to select ADC patients for EGFR mutation analysis, resulting in the included patient population is quite different with actually clinical status. The non-ADC NSCLC patient population accounted for only 77 patients (9.5%) of the 808 NSCLC population in our study population compared with about 38.9% in the actually clinical practice from the Chinese National Office for Cancer Prevention and Control data [24–26]. The pSUV_max_ of non-ADC patients was much higher than that of ADC population in our study (14.30 vs. 9.29, *p* < 0.001) and previously published studies [27, 28]. The EGFR mutant rate of non-ADC NSCLC patients was universally accepted much lower than ADC population. Thus the discriminability of pSUV_max_ for EGFR mutation may be underestimated in our study population as compared to actually clinical practice due to extremely small proportion of non-ADC patients. As commonly applied method to imitate the actually ratio between ADC and non-ADC NSCLC in clinic, the SPSS software enabled us to randomly select 122 ADC patients (16% of those total 731 ADC patients). Then we combined those 122 ADC patients with 77 non-ADC patients for analysis. When pSUV_max_, smoking status, sex and histology of ADC were used together, the AUC of ROC analysis increased from 0.697 to 0.782 (data not shown), which indicated that those four factors could provide relative good performance for predicting EGFR mutation status.

EGFR mutant status from the tissue genetic analysis was the gold standard for first-line treatment with EGFR-TKIs. The guidelines for NSCLC treatment of China [29], ESMO [30], ASCO [31] and NCCN [32] all suggest EGFR mutation testing from tissue assays prior to first-line treatment with EGFR-TKIs. However, EGFR mutation testing was sometimes unavailable for oncologists to decide the therapeutic regimen. In European and North America approximate one-quarter advanced NSCLC patients were not available with EGFR mutation testing in 2015 [33]. In China, EGFR testing rate was only 9.6% in 2010 from a national survey [34], 18.3% in 2011 from nine sites including 12,086 NSCLC patients [35], and 42.5% in 2014 from a non-interventional real world study on EGFR testing in patients with IIIB/IV NSCLC in northern China [36]. An unpublicized investigation from our institution including 4062 NSCLC patients revealed the EGFR mutation testing rate was 45.2% in 2015 and 49.6% in 2016.

Several reasons may account for the low EGFR mutation testing rate in China. First, many patients were not available with tumor tissues. The Health and Social Care Information Centre recorded that about 23% patients were not available with tumor tissues in the United Kingdom [37]. From our clinical experience, about 30% of patients cannot obtain samples owing to personal subjective refusal of invasive examination or several objective reasons resulting in ineligible for sampling including coagulation abnormalities, severe cardiopulmonary insufficiency, arterial and venous pulmonary hypertension, high risk of pneumothorax, usage of anticoagulant drugs and patient uncooperativeness. Second, the diagnosis of NSCLC has been more and more relying on small biopsy or cytologic specimens which however sometimes were not of good quality or sufficient number of tumor cells for EGFR mutation testing. The data from a study concerning “real-world” EGFR mutation testing practices in Asia in 2011 showed that 53.8% sample tested for EGFR mutation were small biopsy or cytology specimens in China [35]. The data from our institute in the last 5 years showed that small biopsy and cytology specimens account for 68.4% of all samples tested for EGFR (data not shown). Minimally invasive examinations for diagnosis of lung cancer included fine-needle aspiration (FNA), core biopsy, bronchoscopy with biopsy and transbronchial needle aspiration (TBNA), endobronchial ultrasound (EBUS)-guided biopsy, and the cytologic specimen from bronchial washing, bronchial brushing, sputum, bronchoalveolar lavage fluid (BALF) and pleural effusion [38]. Those minimal invasive examinations have significantly improved lung cancer diagnosis; however, small samples and cytologic specimens were sometimes not sufficient of quality or quantity for EGFR mutation testing in which the reported failure rates are about 5% to 30% [39–41]. Third, limited medical resource and incomplete implementation of guidelines in some medical institutions may also result in low EGFR mutation testing rate.

Thus, a natural question is how to select the patients potentially benefited from EGFR-TKIs treatment among the patients without available EGFR testing and those who cannot tolerate chemotherapy. Two previous randomized head-to-head clinical trials showed that among patients with unknown EGFR status, patients selected by only clinical factors had a greater response and better PFS with EGFR-TKI treatment than with chemotherapy in the first-line treatment [42–44]. The aim of our study is to investigate whether ^18^F–FDG PET/CT could be a useful modality to enhance patient stratification in some NSCLC patients whose EGFR testing is unavailable. Our result showed that low pSUV_max_ is associated with mutant EGFR status. It could be integrated with other clinical factors to enhance the discriminability on the EGFR mutation status and be used by oncologists to decide the treatment strategy in some NSCLC patients without available EGFR testing.

Different EGFR mutation types can drive distinct downstream signaling, TKI affinities and treatment responses [45, 46]. In-frame deletion in exon 19 and L858R substitution mutation in exon 21 account for most EGFR mutations in NSCLC [47]. A previous study indicated that exon 19 in-frame deletions showed longer PFS following an EGFR TKI treatment [45]. The ^18^F–FDG avidity of these two mutation types was also evaluated previously. Choi et al. showed that the pSUV_max_ of NSCLC patients with the L858R mutation was significantly higher than that of NSCLC patients with the exon 19 mutation (11.6 vs. 8.2) [48]. However, Lee’s group revealed no difference between the two mutation types [22]. Consistent with the result by Lee et al., the pSUV_max_ measurements of the two mutation types were not significantly different in our study.

The EML4-ALK rearrangement is another driver mutation and druggable target in NSCLC. The ALK rearrangement in NSCLC patients shows a dramatic response and prolonged PFS with an ALK TKI treatment [4, 5]. Our study investigated the metabolic features of ALK-positive NSCLC patients. Among the ADC patients, the nSUV_max_ was higher in the ALK-positive group than in the ALK-negative group. However, there was no difference in the pSUV_max_ between the two groups. Jeong et al. reported that a higher pSUV_max_ was an independent predictor of ALK positivity [19]. There are two possible explanations for this discrepancy. First, 53 previously treated patients were included in the study by Jeong et al. Second, selection bias may have influenced the results; the ALK analysis was conducted after evaluating the EGFR and K-ras status, and the ALK positivity (18.6%) of the patient population in the study may have strengthened the statistical significance. Another study that included 5.4% ALK-positive ADCs also showed a higher pSUV_max_ in ALK-positive patients than in ALK-negative patients [20]. These two studies identified ALK-positive patients by FISH, whereas the Ventana IHC system was used to determine the ALK status in our study. The different detection methods may explain the discrepant results. However, the Ventana IHC system is a fully automated IHC assay, with a sensitivity of 100% and specificity of 98%, that has been approved by the US FDA and China FDA for the identification of NSCLC patients who are eligible for treatment with ALK TKIs [49]. Moreover, multiple studies have reported that Ventana ALK IHC is a better predictor of the ALK inhibition outcome than ALK-FISH for advanced NSCLC [50]. The response rates to the ALK inhibitors were 100% in the FISH-negative/IHC-positive cases (7/7) and 46% in the FISH-positive/IHC-negative cases (13/28) [51].

There are several limitations to this study. First, the retrospective design may have introduced bias, including patient selection bias and sample availability bias. Second, the Asia-Pacific region NSCLC/ADC subgroup have the highest EGFR mutation frequency at 47% compared with other regions [52]. The patients in our study were all Chinese and thus had a genetic alteration pattern that was distinct from other races, which may impede the application of our results to other races. Although two studies from Spain and the U.S. also showed lower pSUV_max_ in EGFR mutant NSCLC patients than that in EGFR wild-type patients [11, 12], it is still necessary to note the potential difference between different regions and races. Third, many lymph nodes and distant metastases were not histologically verified. Hence, we did not include nSUV_max_ and mSUV_max_ in the multivariate analysis. Fourth, ^18^F–FDG uptake is actually nonspecific and is a net result of microvasculature for delivering nutrients, glucose transporter of transporting ^18^F–FDG into the cell, HK for entering ^18^F–FDG into glycolysis and the number of tumor cells [53]. Any factors that can regulate those steps will influence ^18^F–FDG uptake. For example, intracellular pH is an important factor influencing glycolysis and ^18^F–FDG uptake [54]. An alkaline intracellular pH could promote glycolysis which depends on the pH-sensitive activity and abundance of several glycolytic enzymes including lactate dehydrogenase [55, 56], phosphofructokinase 1 [57, 58], phosphorylase kinase [56] and fructose-1,6-bisphosphatase [56]. However, different driver mutations may also result in distinct pathways activation and glycolytic features [59–61]. Moreover, driver mutations including K-ras mutation, EGFR mutation, ALK rearrangement, ROS1 rearrangement, PI3K mutation et al. are almost mutually exclusive in NSCLC patients [62]. So it is still reasonable to use ^18^F–FDG uptake to distinguish different driver mutations in NSCLC. A study by Carlos group showed that pSUV_max_ of NSCLC patients with K-ras mutation was much higher than that of NSCLC patitents with EGFR mutation [11].

In conclusion, our study aimed to investigate whether or not ^18^F–FDG PET could be a valuable noninvasive method for predicting EGFR mutations and ALK positivity in NSCLC using the largest patient population to date. We identified that pSUV_mac_ < 7.0 was associated with EGFR mutation in NSCLC patients The AUC of the ROC curve analysis of four factors, including pSUV_max_ < 7.0, female sex, non-smoker status and histologic type of ADC was 0.697. When the ratio between ADC and non-ADC NSCLC patients mimicked the actually clinic status, the AUC increased to 0.782 indicating that those four factors could provide relative good performance for predicting EGFR mutation status. The pSUV_max_ measurements were not different between the ALK-positive and ALK-negative groups in our study. Other noninvasive biomarkers could be investigated and integrated with ^18^F–FDG PET in the future to optimize the predictive power for the EGFR and ALK status when tissues for genetic analysis are unavailable. For example, exquisite algorithms for analyzing diagnostic CTs and PET/CTs have already been developed to obtain more information for predicting genetic alterations in NSCLC patients [16, 17, 63]. Radiolabled EGFR-TKI and anti-EGFR antibody for TKI-PET and immuno-PET are also very promising modalities for predicting EGFR mutation status and clinical efficiency of TKI treatment [64]. Exnograft mouse models and several clinical studies [65–67] have showed exciting results and two clinical trials are ongoing.
